# Hospitalization costs and their determining factors among patients undergoing kidney transplantation: a cross-sectional descriptive study

**DOI:** 10.1590/1516-3180.2018.055117092019

**Published:** 2020-03-06

**Authors:** Maynara Fernanda Carvalho Barreto, Mara Solange Gomes Dellaroza, Karen Barros Parron Fernandes, Paloma de Souza Cavalcante Pissinati, Maria José Quina Galdino, Maria do Carmo Fernandez Lourenço Haddad

**Affiliations:** I RN. Doctoral Student, Universidade Estadual de Londrina, Londrina (PR), Brazil.; II PhD. Nurse and Professor, Nursing Department, Universidade Estadual de Londrina, Londrina (PR), Brazil.; III PhD. Dentist and Professor, Rehabilitation Sciences Program, Universidade do Norte do Paraná, Londrina (PR), Brazil.; IV RN, PhD. Coordinator of Planning, Monitoring and Evaluation of Primary Healthcare Actions in Rolândia, Rolândia (PR), Brazil.; V PhD. Nurse and Professor, Nursing Department, Universidade Estadual do Norte do Paraná, Bandeirantes (PR) Brazil.; VI PhD. Nurse and Professor, Nursing Department, Universidade Estadual de Londrina, Londrina (PR), Brazil.

**Keywords:** Transplants, Kidney, Costs and cost analysis, Kidney transplantation, Hospitalization, Hospital internment, Cost of hospitalization, Transplant complications, Clinical complications

## Abstract

**BACKGROUND:**

Cost evaluation is a key tool in monitoring expenditure for budget management. It increases the efficiency of possible changes through identifying potential savings and estimating the resources required to make such changes. However, there is a lack of knowledge of the total cost of hospitalization up to the clinical outcome, regarding patients admitted for kidney transplantation. Likewise, there is a lack of data on the factors that influence the amounts spent by hospital institutions and healthcare systems.

**OBJECTIVES:**

To describe the costs and determining factors relating to hospitalization of patients undergoing kidney transplantation.

**DESIGN AND SETTING:**

Cross-sectional descriptive study with a quantitative approach based on secondary data from 81 patients who were admitted for kidney transplantation at a leading transplantation center in southern Brazil.

**METHODS:**

The direct costs of healthcare for patients who underwent kidney transplantation were the dependent variable, and included personnel, expenses, third-party services, materials and medicines. The factors that interfered in the cost of the procedure were indirect variables. The items that made up these variables were gathered from the records of the internal transplantation committee and from the electronic medical records. The billing sector provided information on the direct costs per patient.

**RESULTS:**

The estimated total cost of patients’ hospitalization was R$ 1,257,639.11 (US$ 571,010.44). Out of this amount, R$ 1,237,338.31 (US$ 561,793.20) was paid by the Brazilian National Health System and R$ 20,300.80 (US$ 9,217.24) by the transplantation center’s own resources. The highest costs related to the length of hospital stay and clinical complications such as sepsis and pneumonia.

**CONCLUSIONS:**

The costs of hospitalization for kidney transplantation relate to the length of hospital stay and clinical complications.

## INTRODUCTION

Kidney transplantation is considered to be the best therapy for treating chronic kidney disease, which is increasing worryingly in the population.^1^^,^^2^ It has been observed that kidney replacement therapies such as dialysis are costly and that transplantation is the most viable long-term cost-effective procedure.^1^^,^^2^^,^^3^^,^^4^ In addition, kidney transplantation also implies improved quality of life.^5^^,^^6^^,^^7^

The United States is the country in which the highest absolute numbers of kidney transplantations are performed. There were more than 400,000 procedures in the United States between 1988 and 2017.^8^ Brazil is considered to be the country with the second highest absolute numbers of transplantations and has the best transplantation system in the world.^2^ It has been estimated that 6,000 transplantations are performed in Brazil every year, i.e. approximately 30 kidney transplantations per million population.^2^

However, since 2016, the numbers of kidney transplantations performed in many Brazilian states have been declining because the supply of donated organs from deceased donors has been decreasing. The exception to this is the states of Paraná, Rio Grande do Sul and São Paulo, where transplantation rates of more than 45 per million population have been maintained.^9^

For the Brazilian system to remain a global reference, the professionals involved in it need to undertake a chain of successful actions, from identification of potential donors to execution of transplantations and outpatient follow-up.^10^ Faced with the demands of society, managers need to have knowledge of the resources available and the strategies for qualifying patients and expanding access to organ transplants.^11^ Such knowledge contributes towards cost management.

In this context, it is essential to know and evaluate the cost of transplantations from the perspective of public healthcare systems like the Brazilian National Health System. These systems are responsible for the costing of professional and hospital services and, hence, for identifying factors that influence expenditure^12^ and adjusting this to the amounts paid to institutions. Furthermore, such knowledge contributes towards improvement of public policies and adjustment of the amounts paid by public healthcare systems for these procedures. In addition, cost evaluation is a key tool for monitoring expenditure and thus for managing the budget. This increases the efficiency of possible changes through identifying potential savings, estimating the resources required to make such changes and estimating the resources needed to extend the changes.^7^ Research in Japan has recently identified a need to evaluate the cost of patient hospitalization and investigate the impact of clinical complications on the total cost of transplantation.^12^

However, there is a lack of knowledge of the total cost of hospitalization up to the time of the clinical outcomes among patients who are admitted for kidney transplantation, along with the factors that influence the amounts spent by hospital institutions and healthcare systems. Therefore, the aim of this study was to describe the cost and its determining factors relating to hospitalization of patients undergoing kidney transplantation.

## METHODS

This was a cross-sectional descriptive study with a quantitative approach, on the costs and determining factors relating to hospitalization of patients for kidney transplantation. The study was developed in a Brazilian transplantation center, based on secondary data from all patients (n = 81) who had been admitted for kidney transplantation between January 2007 and December 2016. The inclusion criterion was that the transplantations needed to have been funded through the Brazilian public healthcare system.

The institution where this study was conducted is a 335-bed philanthropic hospital that provides services for the Brazilian National Health System. In terms of the complexity and comprehensiveness of the services provided, it is a tertiary-care hospital and is considered to be a type II transplantation center. It has an internal committee responsible for management of transplantations; it emphasizes the role of nurses; and it attends patients from all over the state of Paraná. Furthermore, this institution is a reference center for performing kidney and heart transplantations.

Information was gathered between November 2016 and July 2017. We prepared a spreadsheet, with adjustments after the initial data-gathering, in order to record items from the electronic medical records, data from the internal transplantation committee, records from the institution’s billing sector and information from the remuneration table of the National Health System that was in force and which is updated each year.^13^

The direct costs of healthcare for patients who underwent kidney transplantation were considered to be the dependent variable. The direct costs included personnel, expenses, third-party services, materials and medicines.^14^ They also included, as independent variables, the factors that interfered in the cost of the procedure. The items that made up these variables were gathered from the records of the internal transplantation committee and from the electronic medical records. These were divided into two groups: a) those relating to the patient, such as sex, age, date of admission, date of clinical outcome, clinical diagnosis and complementary clinical information; and b) those relating to the surgical procedure of transplantation, such as type of procedure, type of donor (living or deceased) and surgical complications. The billing sector provided information on the direct costs, along with the cost averages for the days of hospitalization and the estimated values ​​for the surgical procedure and the period of hospitalization for each patient.

In order to measure the direct costs, the study followed the steps and guiding questions proposed by Silva:^7^ definition of the overall proposal of the study, delimitation of the period under study, identification and measurement of costs, definition of the method for deciding cost values ​​and temporal adjustments.

The total amount for hospital service items plus hospital stay values constitutes the total hospitalization cost per patient. The individual and total expenses were described in Brazilian reais (R$) and US dollars (US$), using estimates from quotations made in August 2017. To convert the costs into dollars, the amounts were calculated separately based on the average for each year. To calculate the average quotation for each year, the values from Brazilian financial indices were used. The Emerging Markets Bonds Index Plus, calculated by the United States bank JP Morgan, is used by financial companies for information on country risk.^15^

The data obtained were tabulated in an Excel spreadsheet and were then analyzed using the Statistical Package for the Social Sciences (SPSS) software, version 18.0 (IBM, Chicago, IL, USA). The statistical analysis criteria that were established comprised a 95% confidence interval and a significance level of P ≤ 0.05.

For descriptive analysis on the data, central trend measurements, medians and interquartile ranges were calculated, after the Shapiro-Wilk normality test was firstly done on all the main components. Multiple linear regression was performed to evaluate the influences of the independent variables relating to the patients and the procedures within the hospital costs. These variables were dependent on the model and the analysis.

This study was approved by our institution’s ethics committee for research involving human subjects (procedural no. 1,883,141) on September 28, 2016. Its Brazilian certificate of presentation for ethics assessment (CAAE) number for public consultation is 61063116.0.0000.

## RESULTS

Among the 81 cases of hospitalization that were assessed, 40 admissions (49.4%) were elective and 41 (50.6%) were urgent, for organ transplants from living and deceased donors respectively. Regarding the epidemiological variables, there were more men (54.3%), and the patients’ ages ranged from 12 to 73 years, with an average of 37 years (± 15.29).

Regarding the patients’ previous clinical history and their need for transplants, 69 (85.2%) presented unspecified chronic kidney failure, 12 (14.8%) had end-stage renal disease, 52 (66.7%) were undergoing weekly hemodialysis, 71 (87.7%) had systemic hypertension and 10 (12.3%) had diabetes mellitus. The median waiting time for the surgical procedure after hospital admission was one day, with a range from less than 24 hours (n = 36; 44.4%) to three days.

The total cost of the 81 hospitalizations was R$ 1,257,639.11 (US$ 571,010.44), with a minimum cost per patient of R$ 3,897.27 (US$ 1,769.49) and a maximum of R$ 86,716.58 (US$ 39,372.24). Out of the total cost, R$ 720,198.08 (US $ 326,994.14) comprised expenditure relating to kidney transplantation from living donors and R$ 537,441.02 (US$ 244,016.29) was expenditure relating to deceased donors; R$ 536,828.84 (US$ 243,738.34) was for the surgical procedure, examinations, materials and medicines; and R$ 720,810.39 (US$ 327,336.70) was for the stay in the inpatient unit and intensive care unit.

Table 1 shows the time distribution of hospitalizations and annual costs relating to patients who were discharged from the hospital (n = 77) (the four dead patients are not included). Table 2 shows the amounts paid to the institution for the total cost of hospitalizations through the Brazilian public healthcare system per year, according to the current table of costs and annual adjustments.

**Table 1.** Total cost of hospitalizations and time distribution of patients undergoing kidney transplantation. Paraná, Brazil, 2017

Year	n*	Total cost		Median (P 50%)			Interquartile range 1^st^ (P 25%) to 3^rd^ (P 75%)	
		R$	US$	R$	US$		R$	US$
2007	13	162,157.98	83,239.76	11,545.46	5,926.57	1^st^ 3^rd^	10,147.56 14,327.84	5,209.00 7,354.84
2008	5	103,280.61	56,286.27	15,468.96	8,430.33	1^st^ 3^rd^	9,780.35 34,125.47	5,330.13 18,597.83
2009	8	164,013.40	82,112.75	19,330.61	9,677.80	1^st^ 3^rd^	16,595.57 23,012.90	8,308.51 11,521.33
2010	3	21,453.43	12,187.14	7,899.00	4,487.22	1^st^ 3^rd^	3,897.26 7,899.00	2,213.93 4,487.22
2011	9	87,343.96	52,143.05	9,309.20	5,557.45	1^st^ 3^rd^	7,432.08 12,308.90	4,436.84 7,348.23
2012	6	114,441.73	58,547.94	15,759.15	8,062.32	1^st^ 3^rd^	8,836.04 27,548.80	4,520.48 14,093.86
2013	8	101,923.31	47,237.75	9,907.32	4,591.68	1^st^ 3^rd^	8,047.57 19,850.17	3,729.76 9,199.83
2014	12	147,338.83	62,612.99	11,135.77	4,732.25	1^st^ 3^rd^	8,460,.60 15,852.60	3,595.41 6,736.71
2015	7	144,020.67	43,236.47	20,271.67	6,085.76	1^st^ 3^rd^	15,792.29 23,809.31	4,741.01 7,147.80
2016	6	99,429.51	25,348.86	8,690.34	2,491.14	1^st^ 3^rd^	5,241.82 27,432.10	1,502.60 7,863.58
Total	77	1,145,403.43	479,716.50	11,545.46	60,042.52			

All amounts paid through the National Health System for kidney transplantation procedures from living and deceased donors per year, along with the adjustments made, can be obtained from: http://sigtap.datasus.gov.br/tabela-unificada/app/sec/procedure/display/0505020092/08/2017 and http://sigtap.datasus.gov.br/unitedtable/app/sec/procedure/exhibit/0505020106/08/2017.14.

*Number of patients undergoing kidney transplantation per year. Patients who died (n = 4) were not included because their values were considered to be outliers.

**Table 2.** Total cost paid through the Brazilian public healthcare system and coverage of hospital bills per year. Paraná, Brazil, 2017

Year	n	Total cost paid through the Brazilian public healthcare system		Coverage of hospital bills
		R$	US$	Percentage (%)
2007	13	154,570.70	79,345.03	95.32
2008	5	62,269.77	33,936.01	60.29
2009	8	103,863.50	51,998.91	63.32
2010	3	40,435.73	22,970.50	188.48
2011	9	120,237.50	71,780.02	137.66
2012	6	102,594.50	52,486.94	89.65
2013	8	145,728.80	67,539.99	142.98
2014	14	249,392.90	105,981.81	97.73
2015	8	127,381.30	38,241.16	85.86
2016	7	130,863.70	37,512.88	131.54
Total	81	1,237,338.31	561,793.20	

*All amounts paid through the National Health System for kidney transplantation procedures from living and deceased donors per year, along with the adjustments made, can be obtained from: http://sigtap.datasus.gov.br/tabela-unificada/app/sec/procedure/display/ 0505020092/08/2017 and http://sigtap.datasus.gov.br/unitedtable/app/sec/procedure/exhibit/0505020106/08/2017.^14^

Among the variables that that were found to influence costs through multiple bivariate linear regression (Table 3), multiple linear regression (Table 4) showed that only the lengths of hospital stay in the hospitalization units and in the intensive care unit had any bearing on the costs (P < 0.001).

**Table 3.** Bivariate linear regression model for the cost of hospitalization of patients undergoing kidney transplantation (n = 81). Paraná, Brazil

Variables	B	95% confidence interval	P-value
Age	-98,142	-265,610 to 69,326	0.247
Complications during the hospitalization period	11,126,968	6,634,340 to 15,619,595	< 0.001
Surgical complications	2,920,322	-7,725,767 to 13,566,410	0.587
Duration of ischemia in the transplanted organ	-313,480	-607,500 to -19,461	0.037
Length of stay in the hospitalization unit	552,595	265,472 to 839,717	< 0.001
Length of stay in the intensive care unit	1,014,198	818,191 to 1,210,205	< 0.001
Deceased or living donor	4,896,635	-119,156 to 9.912,426	0.056

B = unstandardized coefficient used in the regression model.

**Table 4.** Multiple linear regression model for the cost of hospitalization of patients undergoing kidney transplantation (n = 81). Paraná, Brazil

Variables	B	95% confidence interval	P-value
Complications during the hospitalization period	11,126.968	1.834,679 to 3.955,611	0.46
Duration of ischemia in the transplanted organ	313,480	171,710 to 348,788	0.49
Length of stay in the hospitalization unit	552,595	506,901 to 829,298	< 0.001
Length of stay in the intensive care unit	1,014,198	955,816 to 1,239,540	< 0.001
Deceased or living donor	4,896,635	10,903,239 to 448,004	0.07

B = unstandardized coefficient used in the regression model.

The mean length of hospitalization (a variable that affects cost) was 17 days (± 11.02), with a range from three to 69 days and a median of 14 days. Out of the total number of patients, 64 had a long stay, consisting of more than 10 days of hospitalization. The mean length of stay in the intensive care unit was seven days (± 8.61), ranging from one to 66 days and the mean length of hospitalization was 10.57 days (± 8.26), ranging from zero to 57 days.

The predominant clinical outcome was hospital discharge with a functioning graft (n = 72; 88.9%), followed by hospital discharge with a nonfunctioning graft (n = 5; 6.2%) and death (n = 4; 4.9%). The total cost of working grafts was R$ 1,046,618.25 (US$ 475,199.87) with a median of R$ 19,103.96 (US$ 8,481.78). The total amount spent on non-functioning transplant hospitalizations was R$ 87,785.16 (US$ 39,857.41) with a median of R$ 18,680.97 (US$ 8,483.46). Among the patients who died, the total cost was R$ 123,235.69 (US$ 55,953.15) with a median of R$ 48,887.71 (US$ 22,196.66).

Regarding the nonfunctioning grafts, the reasons for removal of the grafted organ were intraoperative complications (n ​= 1; 20%) or, during the hospitalization period (n = 4; 80%), organ thrombosis (n = 1), acute organ rejection (n = 2), acute rejection of the organ (n = 2) or healthcare-related infections (n = 1). Among the patients who died, the reasons also related to complications from the surgical procedure and hospitalization, caused by hemorrhagic shock, hypotension, renal ischemia and healthcare-related infections.

Among the 72 patients who were discharged from the hospital with functional grafts, three (4.2%) had intraoperative complications, with a diagnosis of moderate bleeding. The hospital stays of these patients ranged from 22 to 35 days. Out of the other 69 patients, 34 (49.3%) had some type of complication such as healthcare-related infections, sepsis, arterial hypertension, hemorrhage or acute rejection of the donated organ during the hospitalization period. These patients’ length of hospitalization ranged from 13 to 65 days, and their intensive care unit stay from two to 43 days.

## DISCUSSION

This study describes the costs and determining factors relating to hospitalization of patients who underwent kidney transplantation. The results showed that male patients predominated and that the average age among the patients who underwent kidney transplantation was 37 years. Likewise, other studies have found that greater numbers of transplantations were performed on men and that the predominant age group was the adult and elderly public.^16^

The data of the present study also indicated that the numbers of organ transplants from living and deceased donors were similar (40 versus 41) and that the procedures performed varied according to the year in which the patients were operated. Epidemiological information from 2016 showed that there had been a drop of up to 2.4% in the number of kidney transplantations from deceased donors over recent years, while the number of transplants coming from live donors had remained stable.^9^

Through highlighting this information, it can be seen that there is a need for healthcare system managers to develop strategies to increase the number of donors. Some studies have emphasized the importance of implementation of compensation programs for kidney donors in order to increase the number of organs available for transplantation and reduce the costs relating to kidney replacement therapies.^17^^,^^18^ In this case, kidney donors would receive a cash payment or other benefits established through healthcare programs, as recompense for the donated organ.

Regarding the data associated with the various stages of renal illness, comorbidities like arterial hypertension and diabetes mellitus are considered to be the dominant factors in this clinical condition. These comorbidities have also been correlated with longer hospitalization and death^6^^,^^16^ and are reflected in hospitalization costs.^19^

The costs of carrying out hemodialysis prior to transplantation may result in costs that are ​​higher than the cost of kidney transplantation.^3^^,^^4^^,^^5^^,^^7^ Another study conducted in Brazil showed that over the four-year period studied, kidney transplantation from deceased and living donors gave rise to cost reductions per patient of approximately R$ 37,000 and R$ 46,000, in relation to hemodialysis, respectively.^7^

It should be noted that in the present study, it was not possible to identify the cost savings generated through kidney transplantation from deceased and living donors, compared with hemodialysis. This was because there was a lack of information on previous transplantation care and treatment, since most of the patients had been followed up at other healthcare units.

In relation to the values established within the Brazilian public healthcare system, it was necessary to make annual adjustments to the National Health System hospital services (Table 2). This was because of refusal by the transplantation centers to pay for the expenditure on transplants. The annual adjustments served to offset the expenditure borne by these hospital institutions.^13^

To identify the factors that made up the hospital costs, multiple linear regression was used. This showed that the length of the hospital stay was the main factor that influenced the total cost, as had been shown through the results from other studies.^12^^,^^20^ The mean duration of hospitalization was longer than what had been estimated through the National Health System (17 days versus 10 days), including patient admission, surgical procedure and hospital discharge.^13^

The factors that contributed to the length of stay in the intensive care unit and hospitalization units related to the predominant clinical complications after transplantation and to preventable or minor reasons, especially among patients who acquired healthcare-related infections and developed sepsis. A study investigating the total cost of treatment before and after transplantation showed that clinical complications due to urinary tract infection, sepsis or pneumonia had a significant influence on the total transplantation cost and gave rise to long hospital stays. Patients with pneumonia had hospital costs and lengths of hospital stay that were greater than those of patients without clinical complications (50 days versus 44 days; P < 0.01).^12^

The results from the present study, along with those from other studies that investigated the hospital costs of other types of transplantation, such as liver transplantation, showed that complications after transplantation resulted in longer hospital stays, higher daily costs and more use of medication.^20^ Infections are also a major cause of early hospitalization after hospital discharge.^21^ Another study^22^ showed that transplant recipients may be 6.4 times more likely to be hospitalized again after being discharged after transplantation.

The patients’ clinical condition in relation to immunosuppression may also have influenced the outcomes and factors presented here. A study by Taminato^23^ highlighted the challenge involved in management of infectious complications in patients who had received kidney transplantation, due to their compromised immune system. These results demonstrate that there is a need for the healthcare team to develop strategies for prevention of the more common clinical complications after the surgical procedure, such as healthcare-related infections, in order to reduce the costs incurred during hospitalization and after discharge.

Regarding the clinical outcome at discharge, approximately 90% of the patients were discharged with a functioning graft. Over the long term, costs incurred during hospitalization may be lower than those of other surrogate renal therapies, thus indicating the importance of further studies to analyze the efficiency, cost-effectiveness and quality of life of this target population after kidney transplantation.

The present study had limitations in terms of the lack of information in the medical records, even though they were computerized. Since these patients’ treatments are funded through the National Health System, the average cost per day of the stays in the intensive care unit and inpatient units was considered to be a single amount. Moreover, the data referring to the drugs and examinations were made available together, without separating the costs relating to each item.

However, because of the payment policy adopted in the National Health System table of costs, the absence of minor details on each item did not interfere in the study results, given that the coverage of the accounts is for the total values, in packages established for each transplantation procedure.

## CONCLUSION

Over the period from 2007 to 2016, the estimated hospital cost of hospitalization for patients undergoing kidney transplantation was R$ 1,257,639.11 (US$ 571,010.44). Out of this amount, R$ 1,237,338.31 (US$ 561,793.20) was paid through the Brazilian National Health System and R$ 20,300.80 (US$ 9,217.24) from the transplantation center’s own resources. The highest costs related to the length of hospital stay and clinical complications.

The results from this study may help to consolidate the actions of healthcare managers, in order to identify the factors that relate to the cost of hospitalization of patients undergoing kidney transplants, to identify possible strategies for minimizing clinical complications and to stimulate an increase in the number of transplantations performed. It should be added that further studies need to be carried out in order to analyze the patients’ quality of life and the amounts ​​spent on patient follow-up after hospital discharge, especially within the field of supplementary healthcare.
